# Outcome of patients with streptococcal prosthetic joint infections with special reference to rifampicin combinations

**DOI:** 10.1186/s12879-016-1889-0

**Published:** 2016-10-13

**Authors:** E. Fiaux, M. Titecat, O. Robineau, J. Lora-Tamayo, Y. El Samad, M. Etienne, N. Frebourg, N. Blondiaux, B. Brunschweiler, F. Dujardin, E. Beltrand, C. Loiez, V. Cattoir, J. P. Canarelli, C. Hulet, M. Valette, S. Nguyen, F. Caron, H. Migaud, E. Senneville, on behalf of the G4 bone and joint infection study group (G4BJIS)

**Affiliations:** 1 Infectious Diseases Department, University Hospital of Rouen, Rouen, France; 2 Laboratory of Microbiology, University Hospital of Lille, Lille, France; 3 Infectious Diseases Department, Gustave Dron Hospital of Tourcoing, 135 rue du Président Coty, 59200 Tourcoing, France; 4 Unit of Infectious Diseases Hospital 12 de Octubre of Madrid, Madrid, Spain; 5 Infectious Diseases Department, University Hospital of Amiens, Amiens, France; 6 Laboratory of Microbiology, University Hospital of Rouen, Rouen, France; 7 Laboratory of Microbiology, Gustave Dron Hospital of Tourcoing, Tourcoing, France; 8 Orthopaedic Surgery Unit, University Hospital of Amiens, Amiens, France; 9 Orthopaedic Surgery Unit, University Hospital of Rouen, Rouen, France; 10 Orthopaedic Surgery Unit, Gustave Dron Hospital of Tourcoing, Tourcoing, France; 11 Laboratory of Microbiology, University Hospital of Caen, Caen, France; 12 Orthopaedic Surgery Unit, University Hospital of Caen, Caen, France; 13 Orthopaedic Surgery Unit, University Hospital of Lille, Lille, France; 14 French Reference Center for Osteo-Articular Infections (CRIOAC Lille-Tourcoing), Faculty Hospital of Lille, Lille, France

**Keywords:** Rifampicin, *Streptococcus* spp, Risk factors, Prosthetic joint infection, Outcome

## Abstract

**Background:**

Outcome of patients with streptococcal prosthetic joint infections (PJIs) is not well known.

**Methods:**

We performed a retrospective multicenter cohort study that involved patients with total hip/knee prosthetic joint (THP/TKP) infections due to *Streptococcus* spp*.* from 2001 through 2009.

**Results:**

Ninety-five streptococcal PJI episodes (50 THP and 45 TKP) in 87 patients of mean age 69.1 ± 13.7 years met the inclusion criteria. In all, 55 out of 95 cases (57.9 %) were treated with debridement and retention of the infected implants with antibiotic therapy (DAIR). Rifampicin-combinations, including with levofloxacin, were used in 52 (54.7 %) and 28 (29.5 %) cases, respectively. After a mean follow-up period of 895 days (IQR: 395–1649), the remission rate was 70.5 % (67/95). Patients with PJIs due to *S. agalactiae* failed in the same proportion as in the other patients (10/37 (27.1 %) versus 19/58 (32.7 %); *p* = .55). In the univariate analysis, antibiotic monotherapy, DAIR, antibiotic treatments other than rifampicin-combinations, and TKP were all associated with a worse outcome. The only independent variable significantly associated with the patients’ outcomes was the location of the prosthesis (i.e., hip versus knee) (OR = 0.19; 95 % CI 0.04–0.93; *p* value 0.04).

**Conclusions:**

The prognosis of streptococcal PJIs may not be as good as previously reported, especially for patients with an infected total knee arthroplasty. Rifampicin combinations, especially with levofloxacin, appear to be suitable antibiotic regimens for these patients.

## Background

Prosthetic joint infections (PJIs) are uncommon but difficult-to-treat complications that may be life-threatening [1]. *Streptococcus* spp. are responsible for 9 to 10 % of PJIs and represent the second cause of PJIs due to Gram-positive cocci [1]. Previous studies suggested that streptococcal prosthetic joint infections (streptococcal PJIs) are associated with high remission rates [2–4] that are even higher than those observed in staphylococcal PJIs [5]. On the other hand, *Streptococcus agalactiae* (i.e., hemolytic group B streptococci) is generally considered to be associated with a poorer outcome than the other types of streptococcal PJIs, but the reasons for this remain unclear [6]. The optimal antibiotic therapy for streptococcal PJIs is unknown. Penicillins, especially penicillin G and amoxicillin, are first-line options recommended by the Infectious Diseases Society of America (IDSA) guidelines on the management of prosthetic joint infections [7]. However, these agents are unlikely to be effective against bacteria in the stationary growth phase that may be encountered in biofilm infections [8]. The beneficial effect of rifampicin-combinations on the outcome of patients treated for staphylococcal PJIs has been clearly demonstrated, especially in patients treated with debridement, antibiotics, and retention of the implants (debridement, antibiotics, and implant retention; DAIR) [9–12]. However, it is unclear whether these combinations are of interest in cases of removal of the infected implants especially in one-stage exchange where all the bacteria in low-growth phases may not have been totally eliminated during the surgical procedure [13]. Most streptococci are susceptible to both rifampicin and levofloxacin with minimal inhibitory concentration (MIC) inferior to the values obtained in most infected tissues [14, 15]. In contrast with staphylococcal PJIs, the value of rifampicin-fluoroquinolone combinations has never been assessed in both experimental and clinical studies. The value of rifampicin combinations, especially with levofloxacin, for the treatment of streptococcal PJIs has been suggested [16].

Although enterocci and streptococci are not quite similar in terms of virulence and antimicrobial susceptibility, it is however notable that a recent in vitro study showed the superiority of rifampicin-ciprofloxacin combination when compared to amoxicillin or linezolid rifampicin combinations on *Enterococcus faecalis* biofilms formed on plastic [17].

We report herein the results of a retrospective multicenter cohort study that aimed to assess the predictors of the outcome of patients with THP/TKP infections due to *Streptococcus spp.* with special emphasize on the potential benefit of rifampicin-based combinations.

## Methods

### Study design

This was a retrospective, multicenter observational cohort study of patients with streptococcal infection of a total hip/knee prosthetic joint who were followed up in four reference centers in North-West France (Amiens, Caen, Lille-Tourcoing, and Rouen constituting the G4 bone and joint infection study group [G4-BJIS]) from 2001 through 2009.

### Definitions

Prosthetic joint infection (PJI) was defined according to the IDSA guidelines criteria of PJI [7]. The streptococcal origin of PJI was affirmed if ≥ 2 identical *Streptococcus* spp. strains based on the antibiotic susceptibility profile were cultured from valuable samples like joint aspirate, surgical samples, and/or blood cultures. In each case, at least 5 peroperative samples were taken and transported within two hours to the microbiology laboratory. Solid specimens were crushed beforehand by vortexing (in 1 mL of sterile saline solution for 1 min) with sterile glass beads in order to extract bacteria from biofilm. Gram staining was performed for standard samples. After direct examination, standard samples (fluid specimens and tissue homogenate samples) were inoculated onto chocolate agar plus PolyViteX (bioMérieux, Marcy l’Etoile, France), into brain heart broth at 35 °C for 15 days. Sonication of the samples including removed implants was not used. We did not include patients with polymicrobial infections.

Streptococcal PJIs were classified according to the duration from implantation of the prosthesis to the onset of infection as early (<3 months), delayed (>3 months–2 years) and late (>2 years). Hematogenous origin of the infection was suspected in case of late infection with acute (i.e., less than 4 weeks before onset of clinical symptoms and diagnosis of infection) with documented bacteremia.

Remission was defined as the absence of local or systemic signs of implant-related infection at the last contact and the absence of any new surgery or antibiotic therapy related to the streptococcal PJI assessed at least two years after the end of antibiotic treatment. Patients who developed an aseptic loosening that required removal of the prosthesis and for whom per-operative samples were negative were not considered as failures. Treatment failure was defined as any other outcome, including patient death related to the PJI. Relapse or re-infection was determined according to the isolated microorganism.

### Medical and surgical therapy

The present retrospective study was conducted in four different university hospital centers where surgical options and antibiotic treatment strategies applied to patients with PJIs were similar and did not change between 2001 and 2009. DAIR was used in patients with no implant loosening, provided the time from onset of infection and surgical intervention was less than 4 weeks and if soft tissues surrounding the prosthetic site were in good condition. In the other cases, one-stage exchange (1SE) was performed in non-immunocompromised patients with reliable preoperative microbiological information and satisfactory soft tissue. Two-stage exchange (2SE) was preferred for non-immunocompromised patients whose soft tissue was damaged or for whom reliable preoperative bacterial information was unavailable. Arthroplastic resection (AR) was performed in patients for whom joint replacement would not have produced any functional benefit. In all cases treated with DAIR, the mobile parts (polyethylene) of the prosthesis were changed. In cases of 2SE, re-implantation was performed after an antibiotic treatment duration of 6 to 12 weeks with or without an additional antibiotic-free period of 4 weeks and if the C-reactive protein (CRP) value had normalized (i.e., < 10 mg/L), except when chronic inflammatory disease interfered with C-reactive protein values. A gentamicin-loaded antibiotic spacer was systematically used in patients treated with 2SE. After re-implantation of a new prosthesis, the duration of antibiotic therapy depended on results of intraoperative sample cultures (i.e., 2 weeks in case of negative culture results if antibiotic therapy had been stopped at least 2 weeks prior to the intervention, and 6 to 12 weeks in case of positive culture results). New implants were mostly uncemented. Therapeutic strategy was decided for each patient at a multidisciplinary meeting of orthopedic surgeons, infectious disease consultants, microbiologists, and anesthesiologists. In each case, the patient was aware of the different therapeutic options and took part in the final decision. All surgical procedures were performed without antibiotic prophylaxis. A combination of antimicrobial agents administered intravenously was begun intraoperatively immediately after samples were taken. It consisted of a broad spectrum β-lactam agent (e.g., cefotaxime, aztreonam, or imipenem) and a second antimicrobial agent active against methicillin-resistant staphylococci (vancomycin, teicoplanin, or linezolid). This treatment was continued until microbiological results of the preoperative sample culture were available and was then modified based on culture results. Antibiotics were selected based on patient comorbidity and prescribed at doses adapted from those proposed by Zimmerli et al. [1], except for rifampicin, the daily dose of which was 20 mg/kg administered in divided doses given twice a day, without exceeding daily doses of 1800 mg. After discharge from the hospital, the patient was followed up by both the referring surgeon and the infectious disease consultant 1 month after discharge and at the end of antibiotic treatment. The total duration of antimicrobial therapy was 3–6 months, as proposed by Zimmerli et al. [1]. Patients were then followed up by their referring surgeon once annually for a minimum of 2 years. Missing data on patient outcome after the end of antibiotic treatment were obtained by telephone contact with the patient himself/herself or the general practitioner, or when applicable, by reviewing medical records in cases of rehospitalization.

### Clinical parameters

The following data were collected: age, gender, weight, body mass index (BMI), co-morbidities (malnutrition, chronic liver disease, chronic renal disease, the Anaesthesiology Society of America (ASA) score, and fever (i.e., body temperature > 38 °C) assessed at admission for the first septic revision. PJI risk factors such as diabetes mellitus, rheumatoid polyarthritis, immunosuppression, corticosteroids, malnutrition defined as albuminemia under 35 mg/L, previous PJI or previous local surgical intervention, type of surgery, and blood stream infections concomitant to the diagnosis of PJI were also recorded.

### Biological parameters

Biological parameters including blood C-reactive protein and renal and hepatic functions were collected during the episode of infection and during the treatment period. Per-operative specimens were recorded for each patient: number of positive specimens and susceptibility profile to antibiotics. Streptococci yielded from intraoperative sample cultures on standard medium and enriched broth were identified by automated techniques [API® strips (Biomérieux, Marcy l Marcy, France) and VITEK2® cards (Biomérieux, Marcy l Marcy, France). The antimicrobial susceptibility tests were also performed on the VITEK2® automate (Biomérieux, Marcy l, France).

The diffusion agar technique was used in each case, and the procedure and interpretation of the susceptibility tests were performed in accordance with the Comité de l’Antibiogramme de la Société Française de Microbiologie; annual guides from 2001 to 2011) recommendations (http://www.sfm-microbiologie.org).

### Antibiotic regimens

We collected antibiotic regimen, doses, antibiotic treatment duration, and clinical and biological tolerance (side effects) under treatment.

### Statistical analysis

The Pearson χ^2^ test was used to compare qualitative variables and a 2-sample *t* test to compare continuous variables. A *p* value of < .05 was considered to reveal a significant difference. Logistic regression was used to identify independent variables associated with failure. Variables with medical or biological meaning were retained for the multivariate analysis when their effect had a *p* value less than .25. Statistical analysis was performed using STATA, version 7.0 (StataCorp).

### Ethical considerations

All patients’ collected data were anonymized and recorded on a standardized form preventing any personal identification according to procedures defined by the French information protection commission (Commission Nationale de l’Informatique et des Libertés-CNIL); approval from the Institutional Review Board (Espace Ethique) of the Gustave Dron Hospital for the G4-BJIS was obtained.

## Results and discussion

### Population

Ninety-five streptococcal PJI episodes (50 THP and 45 TKP) in 87 patients were identified in our computerized databases. The demographic characteristics and comorbidities of the patients are reported in Table 1. Specifically, thirty-one patients had diabetes mellitus (35.6 %), and 38 (40 %) had an ASA score > 2.

**Table 1:** Demographic characteristics and comorbidities of 87 patients with streptococcal prosthetic joint infections

Variables	No. of patients (%)
Age, years, mean ± SD	69.1 ± 13.7
BMI, kg/m2, mean ± SD	29.9 ± 8.1
Sex ratio, Male/Female	0.85, 40/47
Comorbidities	
Diabetes mellitus	31 (35.6)
Rheumatoid polyarthritis	8 (9.1)
Chronic renal disease	13 (14.9)
Chronic liver disease	9 (10.3)
Malnutrition^a^	25 (28.7)
Neoplasia	7 (8)
Corticosteroids	11 (12.6)
≥ 1 Comorbidity	70 (73.7)
ASA score ≥ 2	38 (40)

*SD* Standard Deviation, *BMI* Body Mass Index, *ASA* Anesthesiology Society of America score

^a^Defined as albuminemia under 35 mg/L

Clinical characteristics of episodes are reported in Table 2. Twenty-one (22.1 %) patients presented with acute symptoms and 19 (20 %) with damaged periprosthetic soft tissue. Most patients (76.9 %) had no previous episodes of septic revisions. The most frequent clinical sign reported at the first septic revision was joint pain in 78 cases (82.9 %), and a sinus tract was recorded in 18 (18.9 %) episodes. Thirty-nine (41.1 %) episodes of PJI were late infections.

**Table 2:** Clinical characteristics of 95 episodes of streptococcal prosthetic joint infections

Characteristics	No. of episodes (%)
Joint	
Hip	50 (52.6)
Knee	45 (47.4)
Type of infection	
Early	31 (32.6)
Delayed	25 (26.3)
Late	39 (41.1)
Hematogenous origin of the infection	18 (18.9)
Clinical presentation	
Pain	78 (82.1)
Fever	52 (54.7)
Sinus tract	18 (18.9)
Acute symptoms	21 (22.1)
Damaged periprosthetic soft tissue	19 (20)
Number of previous surgeries	
0	73 (76.9)
1	21 (22.1)
≥ 2	1 (1.0)

*SD* standard deviation. Definitions of type of infection: early (<1 month), delayed (1 month– ≤ 2 years), and late (>2 years)

### Microbiological results

Among the 95 episodes of streptococcal PJIs, 37 were identified as (38.9 %) group B streptococci, 31 (32.6 %) as viridans group streptococci separate from the milleri-group, 15 (15.8 %) as milleri-group streptococci, and 12 (12.6 %) as β-hemolytic streptococci separate from the group B, including 4 of group A, 2 of group C and 6 of group G.

The proportion of strains susceptible to penicillin G (i.e., minimal inhibitory concentration (MIC) < 0.25 mg/L) was 97.9 % (93/95 tested strains), 91.1 % (61/67 strains) to levofloxacin (i.e., MIC < 1 mg/L) and 100 % (83/83 strains) to rifampicin (i.e., MIC < 0.06 mg/L).

Nineteen (20 %) episodes were associated with concomitant bacteremia due to the same streptococcal strain (i.e., 5 *S. anginosus*, 1 *S. bovis*, 5 *S. constellatus*, 2 *S. mitis*, 2 *S. durans* and 4 *S. oralis*) including 6 episodes of infective endocarditis (i.e., 1 *S. bovis,* 2 *S. mitis*, 2 *S. durans* and 1 *S. oralis*).

The suspected portal of entry was of dental (14.7 %), cutaneous (13.7 %), colic (3.2 %), and gynecologic origin (2.1 %).

### Medical and surgical treatment

Overall, the median delay from the onset of clinical signs of infection and surgical revision was 14 days (interquartile range [IQR]: 5–44). Twenty-six patients (27.4 %) had received systemic antibiotics prior to admission. Surgical options were DAIR (*n* = 55; 57.9 %), 1SE (*n* = 13; 13.7 %), 2SE (*n* = 19; 20 %), and AR (*n* = 8; 8.4 %). In the subgroup of patients treated with DAIR, 21/55 (38.2 %) were operated within 30 days after the implantation and the 34 others between 31 and 90 days; 4 patients treated with DAIR required two debridements.

**Fig. 1:**
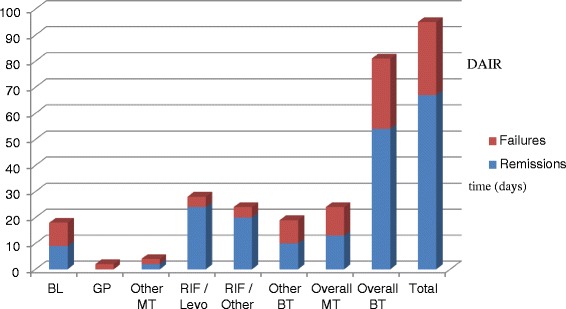
Proportion of failures/remissions of 95 episodes of streptococcal prosthetic joint infections according to the antibiotic regimen. BL (total = 12): Beta-lactam monotherapy (amoxicillin = 14, ceftriaxone = 4). GP: glycopeptide monotherapy (total = 2, all teicoplanin). Other MT: (monotherapy total = 2, all clindamycin). RIF/Levo (total = 28): rifampicin/levofloxacin combination. RIF/Other (total = 24): rifampicin combinations other than rifampicin-levofloxacin (rifampicin and (i) amoxicillin = 12, (ii) trimethoprim-sulfamethoxazole = 5, (iii) linezolid = 3, (iv) teicoplanin = 2, (v) clindamycin = 1, and (vi) doxycycline = 1). Other BT (bitherapy total = 19): other bitherapies (clindamycin-levofloxacin = 14, teicoplanin-ceftriaxone = 3, teicoplanin-levofloxacin = 2). Overall MT (total = 24): antibiotic monotherapies all together. Overall BT (total = 71): antibiotic bitherapies all together

Rifampicin combinations were used in 52 cases (54.7 %), including rifampicin-levofloxacin in 28 cases (29.4 %); 24 patients (25.3 %) were treated with a single agent antibiotic therapy (Fig. 1). Median daily doses of rifampicin and levofloxacin were respectively 1200 mg and 750 mg. The median total duration of antibiotic therapy was 95 days (IQR: 56–121), including a median duration of initial intravenous (IV) administration (i.e., empirical and adapted antibiotic therapy) of 7 days (IQR: 0–15). The median duration of IV antibiotic therapy was slightly longer for patients with concomitant bacteremia and infective endocarditis (16 and 29 days, respectively). Outpatient parenteral antimicrobial therapy was applied in 21 (22.1 %) patients for a median duration of 29 days (IQR: 21–49). Most antibiotics used in this setting were amoxicillin (*n* = 8) and ceftriaxone (*n* = 4). The switch to oral antibiotic treatment was done more quickly in patients treated with rifampicin-levofloxacin combination than in the other patients (mean of 8 versus 12 days, respectively; *p* = .008). No patient required the use of a suppressive antibiotic therapy.

Adverse events compatible with intolerance due to rifampicin combinations therapy were recorded in 17 out of 52 cases (32.7 %), which led to discontinuing the treatment in 5 (9.6 %) cases.

### Outcome

After a median follow-up period of 895 days (IQR: 395–1649), the remission rate was 70.5 % (67/95). Overall, the remission rate of patients with PJIs due to β-hemolytic streptococci was 63.3 % (31/49). Patients with PJIs due to *S. agalactiae* failed in relatively the same proportion as in the other patients (10/37 (27.1 %) versus 19/58 (32.7 %); *p* = .55). Sinus tract or concomitant bacteremia at admission did not influence the patients’ outcome (Table 3). Patients treated with DAIR and a prosthesis age of 0–30 days versus 31–90 days had a similar outcome (13/21 vs 19/34. *p* = .66) and 3 in 4 patients (75 %) with PJIs of haematogenous origin treated with DAIR failed. Overall, patients who received rifampicin combinations including with levofloxacin had a better remission rate than the other patients (Table 4 and Fig. 1). Patients treated with rifampicin-levofloxacin and other rifampicin combinations had comparable remission rates [20/24 (16.7 %) and 16/20 (20 %), respectively; *P* = 0.09)].

**Table 3:** Outcome of 95 episodes of streptococcal prosthetic joint infections; univariate analysis

Variables	Remission (*n* = 67)	Failure (*n* = 28)	*p*
Age > 70 years	35 (36.8 %)	11 (39.3 %)	.25
≥1 comorbidity	46 (68.7 %)	24 (85.7 %)	.09
Total hip arthroplasty	40 (42.1 %)	10 (35.7 %)	.03
Type of infection (early/delayed/late)	20 (29.8 %)/18 (26.9 %)/29 (43.3 %)	11 (39.3 %)/7 (25 %)/10 (35.7 %)	.19
Fever	35 (36.8 %)	17 (60.7 %)	.45
CRP in mg/L, mean value ± SD	154.6 ± 121.9	207.2 ± 148.3	.09
*S. agalactiae* (group B streptococci)	27 (28.4 %)	10 (35.7 %)	.68
Antibiotic treatment prior to admission	18 (18.9 %)	8 (28.6 %)	.86
Sinus tract	15 (15.8 %)	3 (10.7 %)	.18
Concomitant bacteremia at the time of diagnosis	11 (16.4 %)	8 (28.6 %)	.18
DAIR	32 (33.7 %)	23 (82.1 %)	.002
Primary arthroplasty	53 (79.1 %)	20 (71.4 %)	.42
Hematogenous origin	10 (14.9 %)	8 (28.6 %)	.12
Rifampicin based combinations	44 (46.3 %)	8 (28.6 %)	.001
Rifampicin + levofloxacin	24 (25.2 %)	4 (14.3 %)	.04

DAIR: surgical debridement with retention of the fixed components and antibiotic therapy

Results are presented in no. of cases and percentage of the total in each column

**Table 4:** Outcome of 95 episodes of streptococcal prosthetic joint infections according to the type of surgery and the use of rifampicin combinations

Type of surgery	Rifampicin combinations, total = 52	Other antibiotic treatments, total = 43	Total	*p*
DAIR	23/30 (77.7)	9/25 (36)	32/55 (58.2)	.003
1SE	7/8 (87.5)	3/5 (60)	10/13 (76.9)	.25
2SE	10/10 (100)	8/9 (88.9)	18/19 (94.7)	.28
AR	4/4 (100)	3/4 (75)	7/8 (87.5)	.28
Total	44/52 (84.6)	23/43 (53.5)	67/95 (70.5)	.001
Removal	21/22 (95.4)	14/18 (77.8)	35/40 (87.5)	.09

DAIR: debridement, antibiotics, and implant retention

*1SE* one-stage exchange, *2SE* two-stage exchange, *AR* Arthroplastic resection

Removal: 1SE + 2SE + AR

In the univariate analysis, we found five variables associated with a higher risk of failure: DAIR, antibiotic monotherapy, total knee arthroplasty, and antibiotic treatments other than rifampicin or rifampicin-levofloxacin combinations (Table 3). The characteristics of patients who received rifampicin combinations were similar to those who did not, according to the existence of a comorbidity, age of the implant, THP versus TKP, presence of a concomitant bacteremia at admission, and duration of infection before revision (data not shown); the only significant difference between both groups was the mean value of CRP (130.2 ± 113.54 mg/L versus 193.1 ± 140.3 mg/L respectively, *p* = 0.04). When the effect of the use of rifampicin combinations was studied separately according to the surgical option, a favorable effect on the outcome of patients treated with rifampicin combinations only appears for the DAIR option and for the total population of patients (Table 4). When the patients who were treated with removal of the infected implants (i.e., 1/2SE and RA) were studied together, only a trend toward a beneficial effect was observed (*p* = .09; Table 4). We limited the multivariate analysis to the subgroup of 68 patients who underwent either DAIR or one-stage exchange in order to focus on patients for whom rifampicin-combinations are considered an appropriate indication [7]. By doing this, the only independent variable significantly associated with the patients’ outcomes was the location of the prosthesis (i.e., hip versus knee) (OR = 0.19; 95%CI 0.04–0.93; *p* value 0.04). We did not identify any center effect regarding the antibiotic regimens and the surgical options applied to the patients nor for the patients’ outcome (data not shown).

Per-operative samples taken during revisions in each failure patient were positive in 18 out of 28 cases. Failures were assigned to a relapsing infection in 11 cases and to re-infection in 7 other cases. Bacteria identified in failure patients included *Streptococcus* spp, (*n* = 7), coagulase-negative staphylococci (*n* = 5), methicillin sensitive *S. aureus* (*n* = 2), Gram-negative rods (*n* = 3), and *Peptostreptococcus* spp, (*n* = 1). Three of these reinfections were of polymicrobial origin. No rifampicin-resistant strains were identified amongst the 8 failure patients initially treated with rifampicin combinations. During follow-up, 6 deaths—all unrelated to the PJI—were recorded.

## Discussion

To our knowledge, this study is the largest series of patients with PJIs due to *Streptococcus* spp., with more than half of the patients treated with rifampicin combinations, reported so far. Overall, the remission rate of our patients was 70.5 % but failed for 58.2 % (32/55) in the subgroup of patients treated with DAIR, which is lower than reported in the previous studies [5, 18–20]. Zürcher-Pfund et al. reviewed 599 published cases of TKP infections treated with DAIR and found an overall remission rate of 47 % with a significantly higher rate for streptococcal than staphylococcal infection (43/54 (79.6 %) and 144/324 (44.4 %) respectively, *p* < .01)) [18]. More recently, Betz et al. compared the outcome of patients with monomicrobial PJIs treated with DAIR and recorded 0 cases of failure out of 14 cases of streptococcal PJIs versus 19/90 staphylococcal PJIs (*p* = .07) [5]. However, our results are close to those in the study by Sendi et al. who reported a 65 % remission rate in a series of 20 patients with *S. agalactiae* -related PJIS treated with DAIR [19].

As already reported in *S. aureus*-related PJIs, we did not find any difference in the outcomes of patients treated with DAIR within 0–30 days or 31–90 days after implantation of the prosthesis [12]. In this study, multivariate analysis could only identify the location of the prosthesis (i.e., knee prosthesis) as an independent risk factor for failure.

In previous reports of streptococcal PJIs, patients were mostly treated with β-lactam agents, clindamycin, or vancomycin, especially in cases of intolerance to β-lactams [3, 4, 6, 20]. In this study, 52 out of 95 episodes of streptococcal PJIs (54.7 %) were treated with rifampicin combined with another agent especially with levofloxacin in 28 cases. This study provides data regarding the effectiveness and tolerability of rifampicin-fluoroquinolones combinations for the treatment of streptococcal PJIs, which had never been previously reported. We could compare patient outcome according to the antibiotic regimens used as documented treatments in particular because the surgical options used in our patients were comparable in the four G4BJIS investigational centers. The main result established in the univariate analysis is the beneficial effect of rifampicin combinations on the outcome of patients treated with streptococcal PJIS. This effect was only significant in the subgroup of patients treated with DAIR (Table 4), which is consistent with the recent IDSA recommendations for staphylococcal PJIs [7]. Indeed, the benefit of these combinations in patients treated with 1SE has been suggested but has not been clearly demonstrated so far [7]. In addition to the data available for staphylococcal PJIs, a beneficial effect of rifampicin-based combinations has recently been reported by Tornero et al. in patients with acute post-operative enterococcal PJIs [21].

Our patients with *S. agalactiae*-related PJIs failed in the same proportion as the other patients, which differs from the previous study by Zeller et al. [6]. In their study, the authors reported higher failure rates in patients with *S. agalactiae*-related PJIs although the explanations for this remain unknown. The predominant use of rifampicin combinations may explain that our patients had an outcome independent of the streptococcal species involved because rifampicin MICs are very low, irrespective of the streptococcal species [22, 23].

The 58.5 % remission rate obtained in our patients treated with DAIR was significantly lower than that in patients treated with the other surgical options, which is consistent with previous reports [6, 24]. Of note, 15 out of the 55 patients (27.3 %) treated with DAIR had concomitant bacteremia at the time of diagnosis of infection, which may have lowered the remission rate as shown by Vilchez et al. in patients with staphylococcal PJIs [25]. When considering the subgroup of our patients treated with removal of the implants, remission rate achieved is 87.5 % (35/40), comparable to the 94 % value recorded in the series of patients with streptococcal PJIs treated with removal of the infected implants reported by Sendi et al. [19]. The high failure rate recorded in our patients with streptococcal PJIs of haematogenous origin treated with DAIR is consistent with the study from Rodriguez et al. showing a worse outcome of haematogenous PJIs treated with DAIR in comparison to post-operative cases [26].

The rational for using rifampicin-combinations in patients with streptococcal PJIs is limited. Holmberg et al. have shown the beneficial effect of rifampin in an experimental model mimicking PJI due to *Enterococcus* spp., especially in young biofilms [17]. No equivalent studies are currently available for streptococci. However, enterococci are like staphylococci and enterococci Gram positive cocci and it is notable that the beneficial role of fluoroquinolones for the treatment of Gram negative bacilli-related PJIs is admitted without questioning the influence of the type of strain involved, provided it is susceptible to fluoroquinolones.

Rifampicin-based combinations, especially with levofloxacin, allows patients to switch to an oral therapy earlier than for β-lactam therapy due to their high oral bioavailability. The significant number of side effects (32.7 %) reported in our patients treated with rifampicin-levofloxacin combination illustrates the importance of close biological and clinical monitoring of these patients. It must be noted, however, that our patients received high daily doses of rifampicin as recommended in the current French guidelines for the treatment of PJI [27], and this may explain the high rate of adverse events recorded herein, as recently reported by our group [28]. Although levofloxacin exhibits relatively high MICs for streptococci (i.e., around 1 mcg/L), we did not record any cases of acquisition of resistance of streptococcal strains to rifampicin or levofloxacin in our patients with failure. According to our protocol, rifampicin was never administered empirically but exclusively as documented antibiotic therapy consisting of a combination of two agents active against the pathogen(s) identified in reliable samples. The aim of this restriction in rifampicin prescription is to prevent rifampicin monotherapy for *S. aureus* infection, a situation likely to result in the emergence of rifampicin-resistant *S. aureus* mutants [29].

This study has the inherent limitations of its observational retrospective design. In addition, the clonal relationship between streptococcal strains isolated in initial infection and relapses was not determined, which did allow us to precisely evaluate the exact relapse rate in our patients. Finally, the number of patients treated with 1/2SE and AR was low, and the conclusions regarding the absence of beneficial effect of rifampicin combinations in these settings remain to be confirmed in a larger population, although the removal of biofilm bacteria is generally not considered as a good indicator for rifampicin use [7]. Despite these limitations, we think that this study provides useful information for physicians involved in the management of patients with streptococcal PJIs.

## Conclusions

The prognosis of streptococcal PJIs may not be as good as previously reported, especially for patients with an infected total knee arthroplasty. According to our results, *S. agalactiae* PJIs do not appear to be at a higher risk of failure when compared to the other streptococci PJIs. Our results suggest that the outcome of patients with streptococcal PJIs treated with DAIR and who received a rifampicin combination may be better than for any other antibiotic regimens. A multicenter intercontinental retrospective study with a larger sample size is currently underway in order to assess these preliminary results. However, prospective randomized controlled studies would be needed to assess with certainty the role of rifampicin combinations for the treatment of patients with streptococcal PJIs.

## Abbreviations

1SE: One-stage exchange
2SE: Two-stage exchange
AR: Arthroplastic resection
ASA: Anaesthesiology Society of America
BMI: Body mass index
CRP: C-reactive protein
DAIR: Debridement and retention of the infected implants with antibiotic therapy
G4-BJIS: G4 bone and joint infection study group
IDSA: Infectious Diseases Society of America
IQR: Interquartile range
IV: Intravenous
MIC: Minimal inhibitory concentration
OR: Odds ratio
PJIs: Prosthetic joint infections
THP: Total hip prosthetic joint
TKP: Total knee prosthetic joint
